# Tick-Borne Encephalitis Virus Adaptation in Different Host Environments and Existence of Quasispecies

**DOI:** 10.3390/v12080902

**Published:** 2020-08-18

**Authors:** Renata Helmová, Václav Hönig, Hana Tykalová, Martin Palus, Lesley Bell-Sakyi, Libor Grubhoffer

**Affiliations:** 1 Faculty of Science, University of South Bohemia in České Budějovice, 37005 České Budějovice, Czech Republic; Darcenka@seznam.cz (R.H.); tykalova@paru.cas.cz (H.T.); liborex@paru.cas.cz (L.G.); 2 Institute of Parasitology, Biology Centre of the Czech Academy of Sciences, 370 05 České Budějovice, Czech Republic; palus@paru.cas.cz; 3 Department of Virology, Veterinary Research Institute, 62100 Brno, Czech Republic; 4 Institute of Infection, Veterinary and Ecological Sciences, University of Liverpool, Liverpool L3 5RF, UK; L.Bell-Sakyi@liverpool.ac.uk

**Keywords:** TBEV, host alternation, neuroinvasiveness, genome mutation, quasispecies, flavivirus adaptation, tick cell line

## Abstract

A highly virulent strain (Hypr) of tick-borne encephalitis virus (TBEV) was serially subcultured in the mammalian porcine kidney stable (PS) and *Ixodes ricinus* tick (IRE/CTVM19) cell lines, producing three viral variants. These variants exhibited distinct plaque sizes and virulence in a mouse model. Comparing the full-genome sequences of all variants, several nucleotide changes were identified in different genomic regions. Furthermore, different sequential variants were revealed to co-exist within one sample as quasispecies. Interestingly, the above-mentioned nucleotide changes found within the whole genome sequences of the new variants were present alongside the nucleotide sequence of the parental strain, which was represented as a minority quasispecies. These observations further imply that TBEV exists as a heterogeneous population that contains virus variants pre-adapted to reproduction in different environments, probably enabling virus survival in ticks and mammals.

## 1. Introduction

Tick-borne encephalitis virus (TBEV), a member of the genus Flavivirus in the family Flaviviridae, is endemic in many parts of Europe and Asia and causes serious, even fatal encephalitis in humans [1]. AS with other flaviviruses, TBEV is an enveloped virus with single-stranded RNA of positive polarity. The RNA genome of TBEV is about 11 kb in length and encodes a single large polyprotein flanked by 5’ and 3’ untranslated regions (UTR) of variable sizes. Following translation, the viral polyprotein is cleaved by viral and cellular proteases into three structural proteins, namely capsid (C), membrane (M, derived from its precursor prM), and envelope (E) proteins, as well as seven nonstructural proteins, namely NS1, NS2A, NS2B, NS3, NS4A, NS4B, and NS5 [2]. 

In nature, tick-borne flaviviruses are maintained through a transmission cycle involving an ixodid tick vector and a vertebrate host. The virus can persist in ticks throughout their lifespan, enabling virus transmission for years after the initial infection [3]. Although the majority of the evolutionary life of the virus is spent in the tick vector, transmission to a vertebrate host is required to ensure the survival of the virus in natural foci [4,5]. Since arthropod and vertebrate species are only distantly related, flaviviruses have to be very adaptable to persistently infect the arthropod host, yet also to replicate quickly in vertebrates upon transmission. Host alternations presumably select for a virus population that is well adapted to both host systems [6,7]. However, the mechanisms that allow efficient replication in the new host after host switch have not yet been elucidated. 

One possible explanation for the adaptability of RNA viruses and their rapid evolution is the presence of quasispecies. Quasispecies are dynamic distributions of non-identical but closely related mutant and recombinant viral genomes existing as one population in a single host. Quasispecies result from the high error rates of most RNA virus-encoded RNA-dependent RNA polymerases, as well as from short viral generation times and large population sizes [8]. They are subjected to a continuous process of genetic variation, competition, and selection, and act as a unit of selection [9,10,11]. The diversity of viral quasispecies has been shown to be both host- and virus-dependent [12,13], and is a critical determinant of virus fitness [14,15]. A genetically diverse virus population would seem to have an adaptive advantage due to the pre-existence of variants that may have a higher rate of reproduction in a novel or changing environment [16]. The existence of quasispecies was previously described for several mosquito-borne flaviviruses [17,18,19]. There is growing evidence from field studies [16,20] and laboratory experiments [21,22,23] that the same is true for TBEV.

Serial passage of viruses in cell culture in certain cases produces cell-adapted mutant viruses. Many reports state that virus adaptation to cell lines results in reduced virulence in vivo [24,25,26]. However, the mechanism by which cell-adapted flaviviruses undergo attenuation in vivo is unclear. 

In this study, we serially subcultured the highly virulent TBEV strain Hypr in parallel in mammalian porcine kidney stable (PS) cells [27] and in the tick cell line IRE/CTVM19 [23,28], producing three new viral variants. The biological properties of these new variants were investigated in a mouse model and compared to each other, as well as to the parental virus. In addition, complete nucleotide sequences of all these variants were analyzed and differences were appraised as potential genetic determinants important for replication in either the tick or the mammalian host. The correlation between virulence and observed genome changes is discussed. 

## 2. Materials and Methods 

### 2.1. Cell Lines and Viruses

Porcine kidney stable (PS) cells [27] were cultured at 37 °C in L-15 (Leibovitz) medium (PAA Laboratories, Pasching, Austria) supplemented with 3% newborn calf serum (Sigma-Aldrich, Darmstadt, Germany), 2 mM L-glutamine (Sigma-Aldrich, Darmstadt, Germany) and 100 IU/mL penicillin, 100 μg/mL streptomycin, and 0.25 μg/mL amphotericin B (Sigma-Aldrich, Darmstadt, Germany). The tick cell line IRE/CTVM19 [28] derived from *Ixodes ricinus* embryos was grown at 28 °C in L-15 (Leibovitz) medium supplemented with 10% tryptose phosphate broth, 20% foetal bovine serum, 2 mM L-glutamine and 100 IU/mL penicillin, 100 μg/mL streptomycin, and 0.25 μg/mL amphotericin B (Sigma-Aldrich, Darmstadt, Germany). The Czech prototype TBEV strain Hypr was originally isolated from the blood of a 10-year-old child diagnosed with tick-borne encephalitis in 1953 [29]. Subsequently, the strain was propagated through 4 mouse brain passages and used directly as the parental virus strain in our experiments. 

### 2.2. Passage Series and Plaque Size Measurement

The parental virus (designated as 0 P) was serially passaged in PS or IRE/CTVM19 cells forty times, producing two new viral variants (40 PS and 40 IRE). Since TBEV produces permanent infection of the tick cells, a third variant was derived by long-term propagation without passage in tick cells for one month and designated as LT IRE. For a passage series, PS and IRE/CTVM19 cells were seeded in 24-well plates (10^6^ and 10^5^ cells/well respectively) and infected with 10^3^ PFU of the parental virus. The cells were then grown at the respective appropriate temperatures in an atmosphere of 5% CO_2_. After four days of cultivation, the cells were harvested, frozen at −70 °C to release intracellular viral particles, then the suspension was clarified by centrifugation (2500× *g* for 5 min at 4 °C). Subsequently, 100 μL of the supernatant was used as inoculum for the next passage. Plaque morphology and virus titers were determined by plaque assay on PS cells, as described previously [30]. After washing with saline (0.9% NaCl *w*/*v*), the cells were fixed and stained using naphthalene black solution (0.1% naphthalene black in 6% acetic acid solution) for 45 min, subsequently washed with water, then air-dried. During propagation in PS cells, titers of the virus at 4 days post infection fell gradually between passages 20 and 30 due to the increased replication rate of the virus, early onset of cytopathic effect, and thus faster depletion of the cells. Thereafter, the passage interval in PS cells was shortened to two days. 

To assess the evolution of the plaque size during the passaging history, the plaque sizes in passage numbers 5, 10, 20, 30, 35, and 40 in both cell lines and in LT IRE were measured using ImageJ software (NIH, Bethesda, MD, USA, version 1.52a) [31] and compared to the 0 P plaque size. The diameters of a minimum of 20 randomly chosen discrete plaque samples per viral variant were measured in duplicate and mean value sizes were plotted and compared statistically.

### 2.3. Virulence Assays

Virulence assays for all viral variants were performed in adult CD1 mice (TBEV-susceptible strain of mice, females, body weight 15–20 g; AnLab Prague, Czech Republic). Groups of 9 mice were inoculated subcutaneously with 100 PFU of the 0 P, 40 PS, 40 IRE, or LT IRE viruses. Survival rates were recorded daily for a period of 30 days post inoculation (p.i.).

Laboratory animals were used in compliance with all relevant national legislation and regulations of the European Union. The experiments were approved by the Committee on the Ethics of Animal Experiments of the Institute of Parasitology of the Biology Centre of the Czech Academy of Sciences of the Czech Republic.

### 2.4. Virus Replication in the Mouse Model

Groups of 15 adult CD1 mice (females, body weight 15–20 g) were inoculated subcutaneously with 100 PFU of viruses 0 P, 40 PS, or 40 IRE. At different time points p.i., two mice from each group were anesthetized and euthanized. Samples of blood, spleen, and brain were collected. Organs were individually homogenized using a TissueLyser II (Qiagen, Hilden, Germany) and prepared as 20% (spleen) or 33% (brain) (*w*/*v*) suspensions. The suspensions were clarified by centrifugation at 16,000× *g* for 10 min at 4 °C. Blood samples were allowed to clot for 30 min at room temperature and serum samples were obtained by centrifugation at 1000× *g* for 5 min at 4 °C. Samples were analyzed by quantitative reverse transcription–PCR (qRT-PCR).

### 2.5. Quantitative RT-PCR

The number of virus genome copies was determined by qRT-PCR (TaqMan, Waltham, MA, USA). Viral RNA was extracted from serum and organs using a QIAamp Viral RNA Mini Kit (Qiagen). The cDNA was synthesized using a First Strand cDNA Synthesis Kit (Fermentas, Vilnius, Lithuania). Real-time PCR quantitative analysis was performed using the absolute quantification method, whereby the sample concentrations were determined using a standard curve derived from measurements of serial dilutions of a TBEV sample with a known titer. All samples and standards were analyzed in triplicate. The following primers and probe were used: E(F), ACA CGG GAG ACT ATG TTG CCG CA (nt 1409-1431); E(R), CCG TTG GAA GGT GTT CCA CT (nt 1606-1587) [32]; and probe, BHQ1-FAM, ACG CCA CTA GCG ACC CTG CAC AAC A. The qRT-PCR was carried out in a Rotor Gene 3000 instrument (Corbett Research, Cambridge, UK) using an amplification protocol consisting of enzyme activation steps at 95 °C, 10 min; followed by 45 cycles of 95 °C, 15 s denaturation; and 60 °C, 30 s annealing–synthesis steps.

### 2.6. Statistical Analysis

Statistical evaluation of plaque size of TBEV viral variants in comparison to the parental strain Hypr 0 P were tested in GraphPad Prism 8 software using the Kruskal-Wallis test followed by Dunn’s post hoc test corrected for multiple comparisons. Statistical significance of differences in virus growth in sera and organs and the assessment of relative survival rates was evaluated using Statistica (StatSoft CR, Prague, Czech Republic, version 9.). The Fisher’s Least Significant Difference (LSD) post hoc test was used. Statistical significance was accepted at *p* < 0.05.

### 2.7. Genome Analysis

The viral genome was transcribed into cDNA as described above and amplified by PCR using overlapping sets of TBEV-specific primers (Supplementary Table S1) [33]. Sequencing was carried out directly from purified PCR products. Sequencing data were processed using MEGA software, version 4 [34]. The sequences were aligned with ClustalW software in MEGA. Modeling of 3D structures of the modified TBEV E protein was done in GENO3D (http://geno3d-pbil.ibcp.fr) and SwissPdbViewer (Basel, Switzerland, version 4.1.0) [35,36,37] using the E protein structure obtained by crystallography (PDB: 1SVB) [38]. Different model variants were compared using Swiss Model Structure Assessment (http://swissmodel.expasy.org). The best performing model was used for visualization of the amino acid substitutions by DeepView and Swiss-PdbViewer (Basel, Switzerland, version 4.1.0) [39].

### 2.8. Viral Genome Variability

To study viral genome variability within individual isolates and the potential presence of quasispecies, PCR products were cloned using a CloneJET PCR Cloning Kit (Fermentas). Plasmid isolation was performed with a GeneJET^TM^ Plasmid Miniprep Kit (Fermentas). Purified plasmid DNA was then sequenced directly using the pair of universal primers surrounding the vector cloning site.

## 3. Results

### 3.1. Changes in Growth Characteristics During Passaging

**Figure 1:**
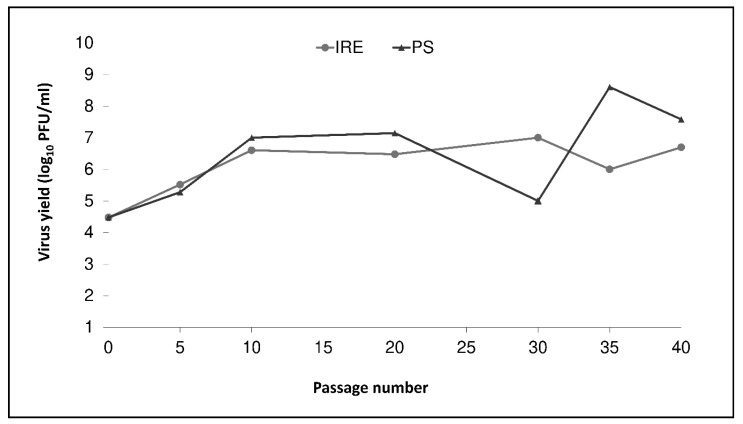
Viral replication kinetics during serial passages of tick-borne encephalitis virus parental strain Hypr in mammalian porcine kidney cells (PS) (dark line with triangles) and in the tick cell line IRE/CTVM19 (light line with circles). The titer was determined using plaque assay and viruses were sampled once at the indicated passage levels per virus variant.

The growth characteristics of TBEV strain Hypr (0 P) were evaluated over 40 serial passages in mammalian (PS) and tick (IRE/CTVM19) cells. Viral titers and plaque sizes of new viral variants were estimated by plaque assay at every 5th passage (except at passage numbers 15 and 25). The titer of the parental virus at the beginning was 3 × 10^4^ PFU/mL (Figure 1). After an initial increase in virus titers in both cell lines, the titer in IRE/CTVM19 cells remained more or less constant, about 10^6^–10^7^ PFU/mL, while the titer in PS cells was more variable, ranging from 10^5^–10^8^ PFU/mL. Between passages 20 and 30, virus replication rates in PS cells increased substantially, resulting in a pronounced cytopathic effect and decrease in the virus titer due to depletion of the host cells at the end of the 4-day incubation period. Thereafter, the passage interval in PS cells was shortened to two days to compensate for the replication rate increase, which led to a subsequent titer increase (Figure 1). 

**Figure 2:**
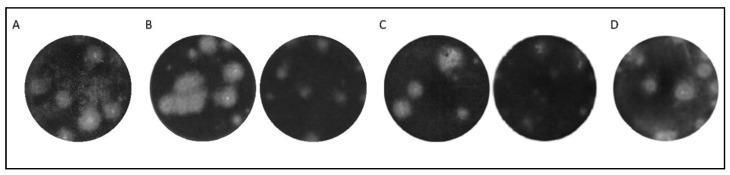
Plaque size and morphology in mammalian porcine kidney (PS) cells during serial passaging of tick-borne encephalitis virus variants in PS and IRE/CTVM19 tick cells. (**A**) Plaque size of the parental strain 0P. (**B**) Difference in plaque size between 30 PS (left) and 30 IRE (right). (**C**) Difference in plaque size between 40 PS (left) and 40 IRE (right). (**D**) Plaque samples of LT IRE after continuous propagation for a month in the IRE/CTVM19 cell line. Well diameter captured in the photographs is 16 mm.

While the plaque size of the virus selected in PS cells did not change dramatically (in 40 PS the mean plaque size was 1.1 mm and over 60% of plaque samples had a diameter above 1.0 mm) in comparison to the parental virus (mean plaque size 1.6 mm and over 60% of plaque samples with diameter above 1.0 mm), the plaque size of the virus selected in IRE/CTVM19 cells changed considerably (Figure 2, Supplementary Figure S1). From the 30th passage, the plaque samples were approximately half the size of the plaque samples of the parental strain (in 30 IRE the mean plaque size was 0.8 mm and more than 70% of plaque samples had a diameter below 1.0 mm) (Figure 2A,B; Supplementary Figure S1). The plaque sizes did not change further up to the 40th passage, which was then used for further analysis (Figure 2C). After the final passage, the derived TBEV variants were designated 40 PS and 40 IRE for the mammalian and tick cell lines, respectively. 

A third experimental TBEV variant (LT IRE) was derived by long term continual propagation in IRE/CTVM19 cells for a period of one month to simulate virus adaptation to permanent infection of tick vectors. From the initial inoculum, the titer of LT IRE increased slightly up to 9 × 10^4^ PFU/mL. In the plaque assay, a mixture of small and large plaque samples was observed (mean plaque size 1.5 mm and 75% of plaque samples had a diameter above 1.0 mm; Figure 2D). The size of the large plaque samples corresponded to the plaque size of the parental virus strain, while small plaque samples corresponded to plaque samples of 30 IRE and 40 IRE variants.

By applying specific cultivation conditions to the TBEV Hypr strain, three virus variants adapted to tick and mammalian cells were derived, exhibiting either altered replication rate in cell culture or altered plaque morphology.

### 3.2. Virulence Assay in the Mouse Model

**Figure 3:**
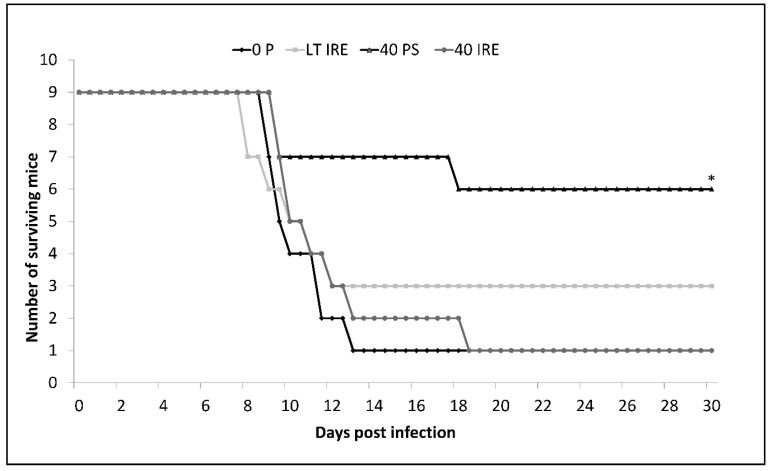
Survival curve of CD1 mice after subcutaneous inoculation with 100 PFU of parental TBEV strain 0 P (dark line with diamonds) or virus variants 40 IRE (light line with circles), 40 PS (dark line with triangles), and LT IRE (light line with squares). Mice infected with 40 PS had significantly prolonged median survival times and lower mortality rates when compared to other viral strains. Statistical significance was tested using Fisher’s Least Significant Difference (LSD) post hoc test (* *p* < 0.05)**.**

Different plaque size of variants obtained under the three different modes of propagation indicated that biological properties of such viruses could vary. To test this hypothesis, we performed a virulence assay in a mouse model. Mice were inoculated subcutaneously with 100 PFU of viral variants or parental strain. A significantly longer median survival time and lower mortality rate were observed in mice inoculated with 40 PS in comparison to 0 P-, 40 IRE-, and LT IRE-inoculated mice (Fisher’s LSD, *p* < 0.05). Moreover, 66% of mice survived the challenge with 40 PS, whereas only 11–33% of mice survived infection with the remaining strains (Figure 3). By challenging laboratory mice with individual TBEV variants, different virulence levels and outcomes of the disease were observed.

### 3.3. Virus Replication in the Mouse Model

To investigate the cause of the differences in the survival rate of mice inoculated with the TBEV variants, the dynamics of virus replication in the mouse model were determined. CD1 mice were challenged with 100 PFU of TBEV parental strain, 40 PS, or 40 IRE variants, then samples were collected daily for eight days from two individuals (except from day 7, when only one individual was sampled). Virus loads in the blood, spleen, and brain were estimated by qRT-PCR (Supplementary Figure S2). In all groups of mice, the virus was first detected in blood 2–3 days p.i., followed by infection of organs (spleen) at days 3–4 p.i. (Supplementary Figure S2A,B). In mice inoculated with 40 PS, a lower level of virus amplification in blood and tissues was observed compared with mice inoculated with 0 P or 40 IRE. However, the viruses differed markedly in their dissemination to the brain. In 0 P- and 40 IRE-infected mice, the virus was present in the brain by the 5th day p.i. and the viral titer increased during the following two days. In mice challenged with 40 PS, the virus was detected for the first time on the 8th day and in one individual only (Supplementary Figure S2C). Due to the limited size of the experimental groups, statistical evaluation of these data was not possible. Results from this experiment are in concordance with the virulence assay results. The viral variant adapted by serial passaging in mammalian cells showed markedly lower neuroinvasiveness than the parental strain or the variant passaged in tick cells. 

### 3.4. Sequence Changes Associated with Adaptation to Mammalian or Tick Cell Lines

To identify genetic changes associated with adaptation to mammalian or tick cell lines and altered neuroinvasiveness in mice, almost the entire viral genome (10,835 bp) was sequenced for the parental virus and for all three new viral variants (40 PS, 40 IRE, and LT IRE). The sequences were submitted to the NCBI GenBank database under the following accession numbers: parental strain: MT228627; 40 PS: MT228628; 40 IRE: MT228625; LT IRE: MT228626. No insertions or deletions were observed in any of the variants. Whole genome sequence analysis revealed 20 single-nucleotide changes, 12 of which were non-conservative at the level of amino acids (Table 1). The highest number of changes compared to the parental virus sequence was recorded in 40 PS (11 nucleotide substitutions, 6 amino acid substitutions). One or more of these substitutions could be responsible for the lower virulence of 40 PS. The 40 IRE variant differed from 0 P in nine nucleotide positions and four amino acids. The least-altered sequence was LT IRE, with only three nucleotide and two amino acid substitutions.

**Figure 4:**
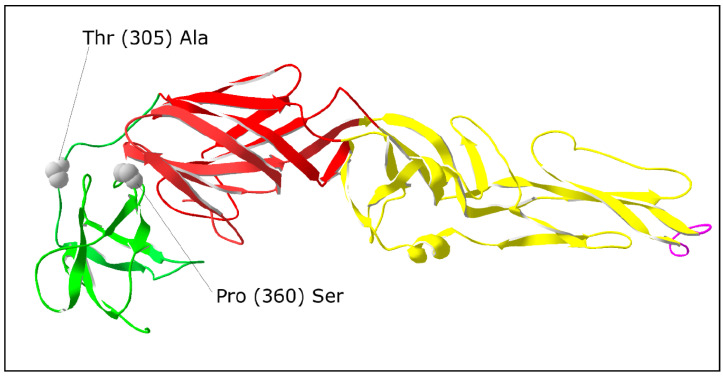
Locations of amino acid substitutions in the TBEV variant 40 PS mapped on the three-dimensional structure of flavivirus E protein (PDB: 1SVB). Domain I—red, domain II—yellow, domain III—green, fusion loop—violet.

To evaluate the potential impacts of nucleotide substitutions on virulence, the locations of the alterations in the least-virulent variant 40 PS was investigated in detail. Amino acid substitutions were found in the NS2A and NS4B proteins (one substitution each) and in the NS5 and E proteins (two substitutions each). Because of several crucial functions of the E protein in the viral life cycle, mutations in this protein were analyzed more closely. The substitution Thr (305) → Ala was situated in the region connecting domain I with domain III (DI–DIII linker). The second substitution Pro (360) → Ser was placed close to the region with a probable function in binding to a cell receptor (Figure 4).

### 3.5. Presence of Quasispecies

**Figure 5:**
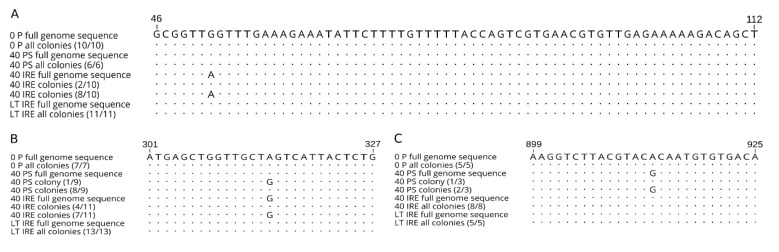
Comparison of partial nucleotide sequences of (**A**) 5’UTR, (**B**) C protein, and (**C**) E protein among clones derived from parental tick-borne encephalitis virus (0 P) and virus variants 40 PS, 40 IRE, and LT IRE. Numbers in parentheses represent the frequency of a particular substitution among the sequenced clones. Dots represent conserved nucleotides, letters indicate substitutions.

Considering the hypothesis of viral adaptation to different environments due to selection from co-existing sequential variants (quasispecies), we tried to identify the sequence variability within our viral variants. Four important parts of the genome were cloned, namely the C protein gene (333 bp), 5’ UTR (132 bp), 3’ UTR (458 bp), and part of the gene coding E protein (796 bp), then between 5 and 13 individual clones per virus variant were sequenced (Figure 5). Nucleotide sequence variability was observed in both coding sequences and the 5’ UTR. On average, the highest nucleotide diversity was observed in the non-coding 5’ UTR (0.4–0.9%). Frequencies of nucleotide changes in the remaining genome regions varied from 0.1% to 0.4% and from 0.3% to 0.4% in C and E protein regions, respectively.

Most of the nucleotide substitutions were found only in a single clone within one viral variant. However, some of the changes were detected in several colonies independently. In the case of position 52 in the 5’ UTR of variant 40 IRE, eight clones carried adenine, while only two carried guanine, as in the parental strain. Interestingly, guanine in this position was observed in all colonies obtained from other viral variants. Consensus nucleotide sequences created from the clone sequences showed 100% identity with the full genome sequence (Figure 5A). Further on in the genome, a synonymous substitution within the C protein sequence of 40 IRE in position 315 was detected. Seven clones had guanine in this position, while in the four remaining colonies adenine was present (Figure 5B). Similarly, variability was found in the E protein sequence of 40 PS in position 913. While this variant had guanine within its consensus genome sequence and sequences of two colonies, a third colony displayed adenine, the same as all colonies of the other viral variants (Figure 5C). To conclude, here we demonstrate the intra-population nucleotide variability contained within individual TBEV variants derived from the parental virus by long-term specific cell line cultivation constraints, which indicates the existence of quasispecies.

## 4. Discussion

Closely related viral strains may produce a considerably different course of infection in the host [40,41]. Detailed information on particular determinants of virulence at the molecular level would allow a better understanding of the infectious process leading to optimization of disease treatment or development of attenuated vaccines. In the current study, by adapting TBEV to different cell lines in vitro, we have obtained strains of TBEV differing in growth characteristics in cell culture, as well as pathogenicity in a mouse model. We attempted to track back the differences in biological properties among these strains to changes at the genome and amino acid levels.

The first apparent difference between the newly prepared variants was in plaque size. While the variant adapted to tick cells (40 IRE) produced plaque samples of approximately half the size of the parental strain, the variant adapted to mammalian cells (40 PS) produced plaque samples of the same size as the parental strain. Similar observations were reported for strains of Siberian subtype TBEV passaged in ticks and tick cell lines [22]. Nevertheless, the assumption that the production of larger plaque samples in cell culture is associated with increased virulence in vertebrates [42] was not confirmed in our virulence experiments in the mouse model. Similar results were obtained in the case of the mutant TBEV Oshima 5–10 strain [24] and related Langat and dengue viruses [43]. Similarly, the small plaque phenotype is not necessarily associated with reduced virulence (neuroinvasiveness) in a vertebrate animal model, as described for the Siberian subtype of TBEV using small plaque purified clones [22].

Frequently described changes related to reduction in TBEV plaque size and attenuation in vivo are mutations in the E protein resulting in an increase of its positive charge, subsequently leading to an increased affinity to glycosaminoglycans, particularly heparan sulphate [21,22,24,25,44]. A combined effect of multiple amino acid substitutions on the small plaque phenotype was suggested previously [45]. Interestingly, no amino acid substitutions were found in the E protein sequence of the 40 IRE that produced a small plaque phenotype. Apparently, different mechanisms may result in small plaque phenotypes (including innate immune responses) [46].

The course of infection with the individual viral variants corresponded to the results of the virulence assay in laboratory mice. The onset of viraemia from the parental strain and viral variants 40 PS and 40 IRE occurred in the blood on days 2–3 after infection, and in the spleen on days 3–4. However, the titer of the 40 PS variant was lower than that of the other two viruses. We speculate that these differences could have been caused by incapacitation of 40 PS in the preceding steps of pathogenesis, either affecting its ability to replicate in the hypodermis [47], infect dendritic cells, or spread to the draining lymph nodes [2,48]. Following replication in the blood and internal organs, TBEV overcomes the hematoencephalic barrier to reach the brain. If the virus is sufficiently neurovirulent, it causes encephalitis [49]. Both of the more virulent variants (0 P and 40 IRE) were detected in mouse brains soon after their presence in blood (by the 5th day p.i.), then their titers rose steeply to the 7th day p.i. The 40 PS variant was first detected in the brain on the 8th day, the last day of the experiment. By comparing the spread of the virus in the blood and tissues to the virulence assay results, we found out that time of death of the mice correlated with high virus burden in the brain. Differences in the degree of virulence between 0 P, 40 IRE, and the attenuated variant 40 PS can be attributed to the lower efficiency of viral replication, demonstrated by the lower level of viraemia, prior to entry into the brain (lower neuroinvasiveness). Lower neuroinvasiveness was also described as a cause of lower virulence in previous studies [24,43].

Full genome sequences were acquired for the three variants of the virus, as well as for the parental strain. The frequency of substitutions was higher in the mammalian cell-line-passaged viruses in comparison to tick-cell-derived variants. This might reflect the faster reproduction cycle in mammalian cells in comparison to tick cells. An increased number of replication cycles gives an error-prone TBEV NS5 viral polymerase a higher chance of introducing a mutation into the genome.

Comparing biological properties, the variant most different from the parental virus was 40 PS. Two amino acid substitutions were found in the E protein. Particular attention was paid to these because of important roles that this protein plays in the viral life cycle. The mutation Thr (305) → Ala was located in the region connecting domain I with domain III (DI–DIII linker). During TBEV entry into the host cell and virion uncoating in the endosome, E protein is exposed to acidic pH, and important conformational changes occur in this region, enabling domain III to take a correct position in the E protein trimer [50]. Therefore, we assume that the mutation, even if it did not affect charge distribution, could influence the spatial interactions between E protein monomers, and consequently all the processes of virion fusion with the endosome membrane and release of viral RNA into the cytoplasm.

Virulence could also possibly be influenced by the second E protein mutation (Pro (360) → Ser) in domain III, since it is close to the region that is supposed to have a role in cell receptor binding. The receptor domain has not yet been exactly defined, and therefore some participation of amino acids in proximity to the receptor binding site cannot be completely excluded. Previously, several single-nucleotide changes that influence virulence were identified in domain III. In the TBEV genome, such changes were found at positions 384 [51], 310 [52], and 368 [53]. All these mutations were associated with lower virulence in mice.

Another amino acid change in 40 PS was Val (202) → Ala in the NS2A protein. This protein is a membrane-associated part of the flavivirus replication complex [54]. NS2A participates in virion assembly and release of infectious particles from host cells. A significant effect on virion assembly was proven for mutations within the restriction site [55] and mutations distorting hydrophobic domains [56]. The mutation in 40 PS was located in the N-terminus, and both original and mutated amino acids were hydrophobic, thus both of the two above-mentioned mechanisms are unlikely to be involved in the viral attenuation observed in the present study. Moreover, the NS2A protein is generally one of the least-conserved proteins in the TBEV genome [57]. Another amino acid change in 40 PS was found in the NS4B protein at position Phe (88) → Ile. NS4B is a transmembrane protein with a poorly defined function that colocalizes with TBEV membrane NS proteins and takes part in replication complex formation and ER membrane invagination [43,58,59]. However, this mutation was possibly related to the adaptation of 40 PS to PS cells. The last two amino acid substitutions were found in the region encoding the NS5 protein, in positions Leu (778) → Phe and Asn (863) → Ser. This highly conserved bifunctional protein works as a methyltransferase and RNA-dependent RNA polymerase [60]. The catalytic domain of the RNA-dependent RNA polymerase lies in position 270–900 and includes six highly conserved regions [61]. Both amino acid changes in 40 PS were found in the catalytic domain of the viral RNA polymerase but outside of conserved regions, so they probably had no influence on its function.

The variant 40 IRE showed almost the same virulence for laboratory mice as the parental virus, even though it differed from 0 P in nine nucleotide substitutions and four amino acid changes. This variant also differed from all the others in terms of plaque morphology. One amino acid mutation was found in position Thr (34) → Ile in the prM protein. This mutation destroyed the only potential glycosylation site in the prM protein, Asn-X-Thr. The prM protein plays an important role as a chaperonin of E protein [62]. Goto and co-workers found that mutations in the glycosylation site of prM caused a considerable decrease of secretion of “virus-like particles” in comparison to the glycosylated variant [63]. Thus, the mutation in prM could cause accumulation of virions in cells, slower viral spread from cell to cell, and consequently smaller plaque samples in PS cell culture. The mutation Ala (85) → Ser found in NS4B could relate to the adaptation of 40 IRE to tick cells. As mentioned before, mutations in this protein possibly participate in adaptation to specific host organisms [43]. The two remaining mutations, Val (57) → Met in the NS1 protein and Val (33) → Met in the NS2B protein, are unlikely to contribute to the phenotype we observed. A mutation in the replication-participating protein NS1 lay neither in any of the 12 conserved cysteines nor in the potential glycosylation site, which is a key region linked to defects of RNA and limited virus production in the case of a related flavivirus [64]. The NS2B protein creates a stable complex with the NS3 protein and serves as a co-factor of NS2B-NS3 serine protease [65]. Position 33 lies outside of the 47 amino acid residues of the central part of the protein involved in this co-factor activity [66].

The variant LT IRE showed a partly attenuated phenotype in comparison with the parental virus. This could have been conferred by two nucleotide substitutions that affected amino acid sequences. The first, Met (471) → Leu, was located in the E protein outside the main ectodomain in a so-called stem–anchor region that participates in binding of E protein to the cell membrane, in interactions with prM protein, and in pH-dependent conformational changes [67]. Position 471 is specifically included in the region serving as protein anchorage to the cell membrane (“anchor”). Some stem–anchor region mutations have been reported previously as a result of TBEV adaptation to tick cells. Mutation at position 426 was responsible for lower virulence for laboratory mice [21]. In another study, mutation at position 496 influenced viral neuroinvasiveness [68]. Thus, it is possible that the mutation at position 471 of E protein conditioned the partial viral attenuation that we observed. The second amino acid change in LT IRE was found in the NS5 protein, in position Thr (177) → Pro, which falls within a functional domain of methyltransferase. Previous studies showed that single-nucleotide mutations in NS5 protein influence viral attenuation, but that these mutations rather participate in the cumulative effect of single mutations, where the biggest influences are from changes in E protein, while mutations in NS5 merely contribute to attenuation [68,69].

Mutations in proteins and in non-coding regions could influence viral pathogenesis [70,71]. In our study, both non-coding regions showed higher nucleotide variability in comparison to coding regions. Untranslated regions participate with their secondary structures in regulation of viral replication, translation, and packaging. Therefore, multibase deletions in particular influence the virulence and viability of the virus [70,71]. However, the extent of attenuation depends on the particular conserved region affected [71]. Previously, single-nucleotide mutations in the 5’ UTR related to the production of smaller plaque samples have been described [21,72]. Therefore, it is possible that mutation G (52) → A in 40 IRE resulted in the production of small plaque samples, the only phenotypic trait where this variant differed from all the others.

The T (282) → C mutation in the 3’ UTR was shared by 40 PS and 40 IRE variants, which differed in plaque size and virulence in vivo. Involvement of this particular mutation in newly acquired phenotypic traits in these variants is, thus, unclear. This mutation lies in the terminal 190 nucleotide region that forms the conserved 3’stem loop. This secondary structure is required for viral RNA cyclization and replication, and was also identified as an important determinant of virulence [73]. In the related West Nile virus, single-nucleotide mutations in a region responsible for cyclization have impaired the replication efficiency of the virus or plaque size [74]. However, the specific cyclization sequence in TBEV has not yet been defined. Thus, the implications of our findings cannot be confirmed without further investigation.

In summary, both amino acid changes in the E protein of the 40 PS probably contribute to lower virulence in vivo, while the mutation in the NS4B protein most likely arose as a consequence of viral adaptation to PS cells. An underlying role of the structural genes in the pathogenicity for mice was reported previously [75]. Small plaque production by 40 IRE might be the consequence of mutations in the prM protein and 5’ UTR, while the mutation in the NS4B protein arose most likely as a consequence of viral adaptation to tick cells. In order to determine the exact contribution of each of these amino acid changes to the virus phenotype, each of the mutations observed in this study should be investigated individually or in combination using mutated TBEV infectious clones. Results from several studies dealing with attenuated viral strains indicate that viral adaptation to a specific environment does not happen only on the basis of actual random mutations. It is more likely that the adapted variant is selected from an already existing set of sequential variants—quasispecies [16,21,22,23,76]. To explore the variability within the individual viral variants, we used cloning and sequencing of parts of the 5’ UTR, E protein, and C protein, focusing on nucleotide variability in positions in which we documented changes by whole genome sequencing. Several independent clones were used to minimize the possibility of accidental nucleotide substitutions caused by the error rate of Taq polymerase during PCR amplification [77]. Several mutations were found in more than one colony per viral variant.

The mutation (G (52) → A) in the 5’ UTR and the non-conservative mutation in the E protein (Thr (305) → Ala) may play roles in virus adaptation to different environments. In both cases, the same situation was observed. In the viral variant in which nucleotide variability was detected, the minor base was identical to whole genome sequences of the remaining viral variants, as well as to all their colonies. Very similar results were obtained with the Langat virus [43], West Nile virus [18], and the Siberian subtype of TBEV [22]. Thus, it is possible that after the emergence of a new mutation that is advantageous in a new environment, the original variant is retained by a certain mechanism, albeit at a lower frequency. This assumption corresponds with results of previous experiments, in which new viral variants of TBEV were obtained after serial passaging in ticks or tick cell lines, which produced a mixture of small plaque samples and plaque samples of original size in mammalian cell culture. After plaque purification and sequencing, nucleotide changes unique to the virus producing small plaque samples and to phenotypic revertants were detected [21,22]. Phenotypic variability after serial passaging of TBEV in ticks was also observed in the study by Labuda and co-workers [76]. In another study [78], two virulent variants were obtained from a naturally attenuated TBEV strain after five passages in mice or a single passage in PS cells. These variants had identical nucleotide substitutions in their genomes.

Unfortunately, we cannot be sure which of the mutations found in our study provides a selective advantage within a certain environment and which are just equivalent alternatives without any influence on viral traits. The mutation in the E protein, causing the change of amino acid in the connection of domains I and III, probably influences the protein function, as discussed above. The mutation in the 5’ UTR seems to have some importance because the ratio among the clones (8:2) shows strong dominance of A over G. The direct relationship of a single-nucleotide change in the 5’ UTR to the change in phenotypic traits, specifically to plaque size, is less probable, as other authors emphasize the cumulative effect of single-nucleotide changes and their combinations on viral traits [43,45,79]. The substitutions found in our study confirm some changes from a previously published list of mutations involved in changes in virulence and other biological properties of TBEV and other flaviviruses [73], and even contribute further evidence of such involvement. Such information may be particularly important for genetic comparisons with sequences acquired from newly isolated TBEV field strains and viruses sequenced directly from ticks or clinical samples.

To conclude, serial passaging and long-term persistent infection of tick cell lines do not result in attenuation of TBEV in a vertebrate host. Plaque size in mammalian cells is not directly linked to the virulence of a viral strain. There are mechanisms ensuring maintenance of a certain level of genotypic and phenotypic variability during an adaptation process, which allow rapid selection of adapted variants from a pre-existing pool of viral variants (quasispecies).

## Figures and Tables

**Table 1:** Genetic differences between the parental tick-borne encephalitis virus (0 P) and individual new variants (40 PS, 40 IRE, and LT IRE) based on a comparison of full genome sequences. (UTR stands for “untranslated regions”)

Genome Region	Nucleotide Substitution		Amino Acid Substitution	
	Substitution	TBEV Variant	Substitution	TBEV Variant
5′ UTR	G (52) → A	40 IRE		
Protein C	A (315) → G	40 IRE		
Protein prM	C (101) → T	40 IRE	Thr (34) → Ile	40 IRE
Protein E	A (913) → G	40 PS	Thr (305) → Ala	40 PS
	C (1078) → T	40 PS	Pro (360) → Ser	40 PS
	A (1411) → T	LT IRE	Met (471) →Leu	LT IRE
Protein NS1	G (169) → A	40 IRE	Val (57) → Ile	40 IRE
	G (237) → A	40 PS		
Protein NS2A	T (605) → C	40 PS	Val (202) → Ala	40 PS
Protein NS2B	G (97) → A	40 IRE	Val (33) → Met	40 IRE
Protein NS3	T (978) → C	40 PS, 40 IRE		
	A (1314) → G	LT IRE		
Protein NS4B	C (240) → T	40 PS		
	G (253) → T	40 IRE	Ala (85) → Ser	40 IRE
	T (262) → A	40 PS	Phe (88) → Ile	40 PS
Protein NS5	G (333) → A	40 PS, 40 IRE		
	A (529) → C	LT IRE	Thr (177) → Pro	LT IRE
	C (2332) → T	40 PS	Leu (778) → Phe	40 PS
	A (2588) → G	40 PS	Asn (863) → Ser	40 PS
3′ UTR	T (282) → C	40 PS, 40 IRE		

## Supplementary Materials

The following are available online at https://www.mdpi.com/1999-4915/12/8/902/s1: Table S1: List of sequencing primers used to amplify overlapping regions of the TBEV genome. Figure S1: TBEV plaque size distribution during passages in porcine kidney stable (PS) and tick (IRE/CTVM19) cells. Figure S2: Replication of mammalian-cell- and tick-cell-derived variants of TBEV in mice.
