# Supplementary material for: Tick-Borne Encephalitis Virus Adaptation in Different Host Environments and Existence of Quasispecies
Source: Viruses. 2020 Aug 18;12(8):902. doi: 10.3390/v12080902 (PMC7472235; doi:10.3390/v12080902)
Supplement: Supplementary file 1 [file viruses-12-00902-s001.zip › viruses-887041-supplementary.pdf]

Supplementary Material

Supplementary Table S1: List of sequencing primers used to amplify overlapping regions of the TBEV genome.

Primer name	Polarity*	Nucleotide Sequence 5´->3´	Annealing Temperature [°C]	Genome Position **
1C	F	AGA TTT TCT TGC ACG T	48	1-16
1D	R	TTC CTT CAG CTC TGA TC	48	532-516
2C	F	GGT CAT TAC TCT GTT GGG GA	45	450-469
2D	R	ACA CAT CCA TTG AAG GCT TC	45	1102-1083
1A	F	TGT CTA CGC TTC GCG TTG CAC ACA	55	963-986
1B	R	GGA ACC CCA GCG AGA GCC TTC AGT A	55	1787-1763
2A	F	CGT GTT GAA TTT GGG GCT CCT CAC G	54	1693-1717
2B	R	TTC GTT CCG TGT CCA CAG CGC A	54	2491-2470
1M	F	GCT TTC AAC AGC ATC TTC GG	49	2302-2321
1N	R	TGT TAT ACC AGG ATC TGG GTC C	49	3229-3208
2M	F	ACT TTT GGT CAC TGA CCT GAG G	49	3105-3126
2N	R	TCC ATG ATG CAC AGT CAC CA	49	4032-4013
1O	F	GCT GGT CCT GGA ATT GGG GC	52.8	3855-3874
1P	R	GGA CCG GCC ACA GCA TCA TC	52.8	4808-4789
2O	F	GGT CGA GAG CGT GGT GAT AG	50.8	4627-4646
2P	R	CAG GAG GTG TCG CTG TCA TC	50.8	5575-5556
1R	F	CCT ATG TCA ACA GAC GGC TAC T	50	5408-5429
1S	R	GGC TCC TAA AGG TAA CCA AGT C	50	6358-6337
2R	F	GCC CGT GGC CAC CTT CTA TG	52.8	6114-6133
2S	R	CCC CCC GTT CAC TCC ACA AC	52.8	6976-6957
1K	F	CCG AGG CGG GAA AAC AGA GA	50.8	6821-6840
1L	R	AGG ATG CCA GTG CGC CTG TA	50.8	7763-7744
TBE-7547 (K2)***	F	CTG ACA CGT TGT GGA CGA TG	50	7547-7566
TBE-c8732 (K7)***	R	AAC ACT CTC TGC TGT CCG AAA G	50	8732-8711
TBE-8641 (K3)***	F	GTG AAA CTT CTC AGC TGG CC	42	8641-8660
TBE-c9685 (K8)***	R	TTG CCA AAT CTG TCA TCC	42	9695-9678
TBE-9244 (K4)***	F	AAT GGA GGA CTC TTC TAT GCA G	49	9244-9265
TBE-c10140 (K6)***	R	CAT GAA AGG GTT GTC CAG AAT C	49	10140-10119
TBE-2E hypr	F	TTG TTC ACA CCA CTT CCA CG	47	9792-9811
TBE-2T hypr	F	CGT TGG GAG GAA AGA AAG AG	47	10218-10237
1F	R	AGC GGG TGT TTT TCC GAG TC	47	10835-10816

* F—forward primer, R—reverse primer. ** according to the genome of TBEV strain Hypr (gb: U39292). *** Kupča *et al.*, 2010 - modified.

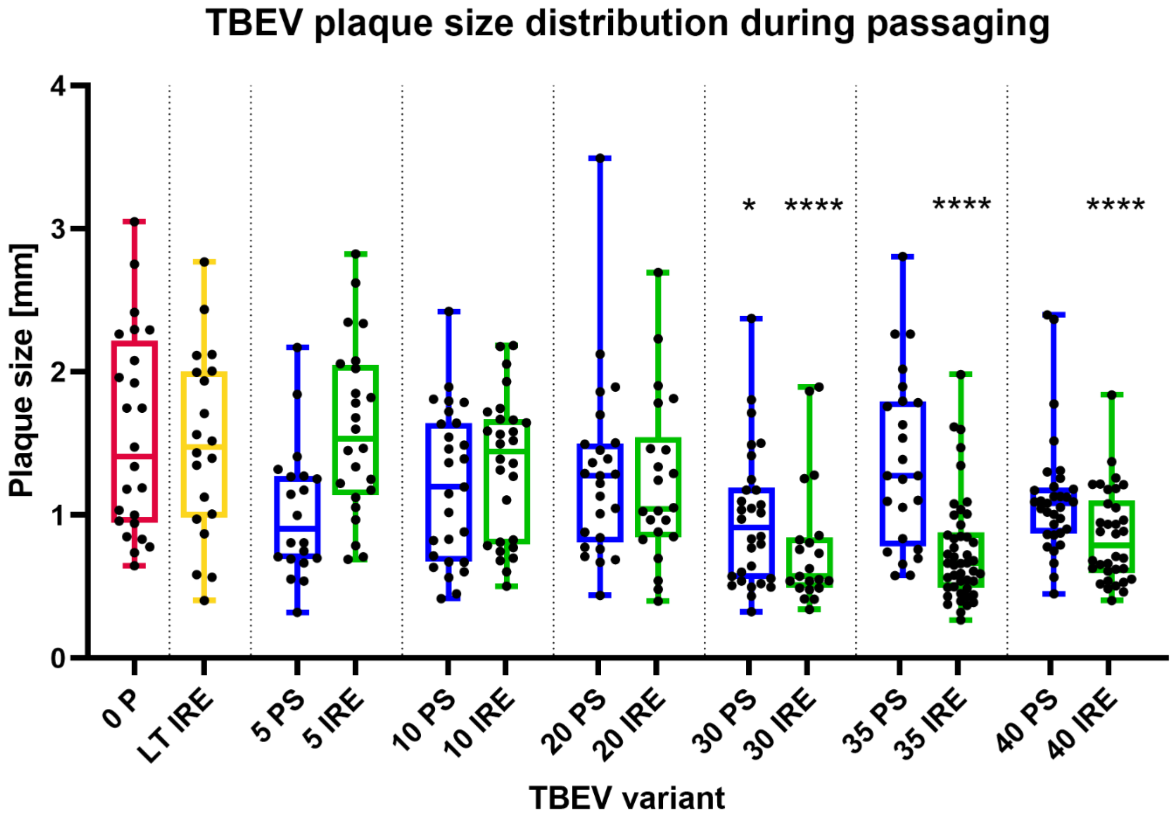

Supplementary Figure S1. TBEV plaque size distribution during passages in porcine kidney stable (PS) and tick (IRE/CTVM19) cells. The Hypr strain of TBEV (0 P; red) was passaged sequentially 5, 10, 20, 30, 35 and 40 times in PS (blue) or IRE/CTVM19 (green) cell lines, giving rise to the viral variants 5 PS – 40 PS and 5 IRE – 40 IRE respectively, or maintained continuously for a month-long period without passaging in IRE/CTVM19 (yellow) cells producing the LT IRE variant. The diameters of a minimum of 20 (range 20-46) randomly-chosen discrete plaques per viral variant were measured using ImageJ software in duplicate and mean value sizes were plotted as a box plot. The middle line indicates the median, the hinges correspond to the 25th-75th percentiles, the whiskers extend to minimum and maximum values measured, and the points represent individual plaques. Statistical evaluation of plaque size in comparison to the parental strain Hypr 0 P was done in GraphPad Prism 8 software using Kruskal-Wallis test followed by Dunn´s post hoc test corrected for multiple comparisons; statistically-significant differences are indicated by asterisks (* <0.05, **** <0.0001).

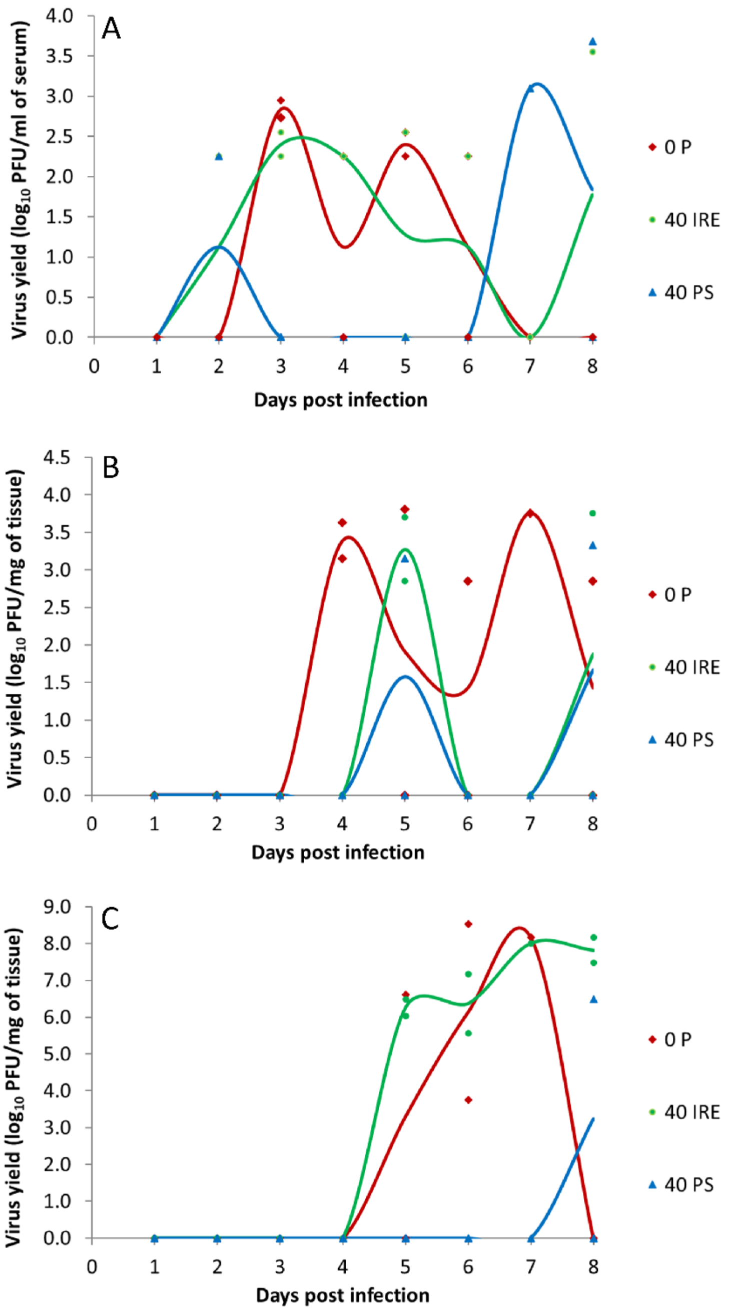

Supplementary Figure S2. Replication of mammalian cell- and tick cell-derived variants of TBEV in mice. Female CD1 mice were infected subcutaneously with 100 PFU of either parental TBEV strain 0P (red and diamonds) or derived viral variants 40 PS (blue and triangles) or 40 IRE (green and circles). The amount of viral RNA retrieved in (A) serum, (B) spleen, (C) brain was determined by quantitative RT-PCR. The calibration curve for qRT-PCR was based on determination of the viral titre by plaque assay and results are thus expressed as $\log_{10}$ PFU/ml of serum or PFU/g of tissue. Individual values are indicated by symbols, lines represent mean values (two mice were sampled per day except for day 7 p.i. when only one mouse was sampled) and show the indicative course of infection for easier evaluation of the data. Samples and standards were analyzed in technical triplicates.
